# Association between urea trajectory and protein dose in critically ill adults: a secondary exploratory analysis of the effort protein trial (RE-EFFORT)

**DOI:** 10.1186/s13054-024-04799-1

**Published:** 2024-01-16

**Authors:** Ryan W. Haines, John R. Prowle, Andrew Day, Danielle E. Bear, Daren K. Heyland, Zudin Puthucheary

**Affiliations:** 1 Adult Critical Care Unit, The Royal London Hospital, Barts Health NHS Trust, Whitechapel Road, London, E1 1BB UK; 2 William Harvey Research Institute, Queen Mary University of London, London, UK; 3 Department of Renal Medicine and Transplantation, The Royal London Hospital, Barts Health NHS Trust, Whitechapel Road, London, E1 1BB UK; 4 Clinical Evaluation Research Unit, Kingston Health Science Center, Kingston, ON Canada; 5 Departments of Critical Care and Nutrition and Dietetics, Guy’s and St. Thomas’ NHS Foundation Trust, London, UK; 6 Department of Critical Care Medicine, Queen’s University, Kingston, ON Canada

**Keywords:** Urea, Multi-organ failure, Intensive care, Protein, Joint modelling

## Abstract

**Background:**

Delivering higher doses of protein to mechanically ventilated critically ill patients did not improve patient outcomes and may have caused harm. Longitudinal urea measurements could provide additional information about the treatment effect of higher protein doses. We hypothesised that higher urea values over time could explain the potential harmful treatment effects of higher doses of protein.

**Methods:**

We conducted a reanalysis of a randomised controlled trial of higher protein doses in critical illness (EFFORT Protein). We applied Bayesian joint models to estimate the strength of association of urea with 30-day survival and understand the treatment effect of higher protein doses.

**Results:**

Of the 1301 patients included in EFFORT Protein, 1277 were included in this analysis. There were 344 deaths at 30 days post-randomisation. By day 6, median urea was 2.1 mmol/L higher in the high protein group (95% CI 1.1–3.2), increasing to 3.0 mmol/L (95% CI 1.3–4.7) by day 12. A twofold rise in urea was associated with an increased risk of death at 30 days (hazard ratio 1.34, 95% credible interval 1.21–1.48), following adjustment of baseline characteristics including age, illness severity, renal replacement therapy, and presence of AKI. This association persisted over the duration of 30-day follow-up and in models adjusting for evolution of organ failure over time.

**Conclusions:**

The increased risk of death in patients randomised to a higher protein dose in the EFFORT Protein trial was estimated to be mediated by increased urea cycle activity, of which serum urea is a biological signature. Serum urea should be taken into consideration when initiating and continuing protein delivery in critically ill patients.

*ClinicalTrials.gov Identifier*: NCT03160547 (2017-05-17).

**Supplementary Information:**

The online version contains supplementary material available at 10.1186/s13054-024-04799-1.

## Introduction

Establishing the optimal dose of protein has been identified as a research priority in the field of critical care nutrition to inform clinical practice [1]. While protein is a fundamental requirement for stimulation of protein synthesis and cellular function and survival, the current evidence remains insufficient to recommend an optimum dose. Observational studies are conflicting with some leading to hypotheses that higher doses of protein in the critically patient may lead to harm [2]. In an 85 centre study of 1301 patients, the EFFORT Protein trial randomised patients to 2.2 g/kg/day or more of protein versus 1.2 g/kg/day or less for 28 days [3]. No benefit was seen from higher doses of protein, with a signal for potential harm in a cohort of ICU patients with multi-organ failure. While the association between protein load and adverse clinical outcomes in patients with multi-organ failure has been previously hypothesised [4], the EFFORT Protein data highlights the need for this association to be investigated in detail, to identify which patients may be harmed by high protein dose. Randomised controlled trials estimate the overall effect of an intervention, but in their delivery, important data on the mechanism through which this intervention exerts its effect may also be collected [5]. We are increasingly aware that heterogeneous responses to interventions are seen in critically ill patients as a result of diverse baseline and disease-related factors [6]. Identifying biological signatures of effectiveness (or harm) via mechanistic understandings of these interventions could aid the future population enrichment for subsequent studies [7]. Applying joint modelling to longitudinal data collected from clinical trials may address this, and in the case of EFFORT Protein distinguish and isolate the effects of ureagenesis from protein loading on survival [8, 9]. Serum urea increases have consistently been observed across the intervention arms of nutritional RCTs of amino acid supplementation [10, 11]. The capacity for the critically ill patient to use amino acids for muscle protein synthesis is estimated to be 60% lower than that of a healthy human [12].

Protein not taken up by tissues are degraded to amino acids and further metabolised to ammonia and thereafter into urea. The rate limiting step is carbamoyl phosphate synthetase 1, which is ATP dependent and may therefore be limited by altered substrate utilisation [13, 14]. While ammonia is toxic, its measurement is not recommended in the majority of clinical settings [15]. Further serum ammonia levels show variability from sample handling and processing, making multicentre evaluations unreliable [16]. Urea acts as a biological signature of urea cycle function and ammonia disposal, and can be measured at scale, e.g. in an 85 centre trial randomising 1301 patients. There is both biological rationale and observational data for ureagenesis as a result of altered or excess urea cycle activity in multi-organ failure [17].

We hypothesise that there is a sequential causal relationship between higher protein delivery, increased urea cycle flux and increased risk of death, with urea acting as a biological signature of toxic protein deamination products. Effective randomisation to higher urea production allows the effect of ureagenesis to be specifically examined. We aimed to estimate this via a joint model of the time-to-event outcome (30-day mortality) and a longitudinal maker (urea) using data from EFFORT Protein. This data will add to the increasing body of evidence supporting targeting of routinely collected data to guide nutritional therapy, aiming to reduce harmful metabolic effects and, ultimately, improve patient outcomes.

## Methods

### Study design

Exploratory secondary analysis of a randomised controlled trial based on STROBE guidance.

### The EFFORT protein trial

A multicentre, pragmatic, volunteer-driven, registry-based, randomised, open-label, clinical trial comparing a higher protein dose (> = 2.2 g/kg/day) to a lower (< = 1.2 g/kg/day) on time-to-discharge alive from hospital and 60-day mortality. The trial included 1301 adult (> = 18 years) participants with nutrition risk and requiring mechanical ventilation for > 48 h.[3, 18] Patients who did not receive the study intervention had missing hospital outcomes or only had one urea measurement were excluded from this post-hoc analysis.

### Approvals

The EFFORT Protein trial is registered at clinicaltrials.gov (NCT03160547—2017-05-17). No core funding was received for this reanalysis. The investigator-initiated EFFORT Protein trial protocol was approved by the Research Ethics Committees of Queen’s University, Canada, and a central institutional review board (IRB) at Vanderbilt University, Nashville, Tennessee, USA that granted waiver of informed consent for sites that acceded to this central IRB. Otherwise, where required by local study sites, local ethics approval was obtained, and informed consent was also obtained from designated patient surrogates before randomisation. All sites and personnel that participated in the data collection are listed in the Supplementary Appendix of the main manuscript [3].

### Patients

We considered all 1301 patients of the modified intention to treat analysis of the primary EFFORT Protein trial [3].

### Procedures and outcomes

Baseline and daily data collection for EFFORT Protein are outlined in detail in the trial protocol [18]. Urea measurements were collected for 12 days. Persistent organ dysfunction (POD) was scored daily for 30 days for receipt of: mechanical ventilation, vasopressor therapy, and renal replacement therapy. Covariables included the treatment effect of higher protein and the following a priori baseline covariates:

- Age; increasing age is associated with increased illness severity and potential increase in inability to process excess protein load [19].
- Illness severity (SOFA score at baseline); increased severity of illness may affect the capability to process excess protein, with more severely ill patients then experiencing the negative effects of mal-processed amino acids [17].
- Acute kidney injury (at enrolment); kidney function and kidney support may affect the metabolic impact of higher protein doses. Some observational data have suggested harm from amino acid delivery in patients with AKI. [20–22]
- Renal replacement therapy (RRT; administered on the day of enrolment). There is an increased risk of mortality in patients receiving RRT while extracorporeal clearance of urea alters longitudinal urea trajectory [17].

The co-primary outcome was 30-day mortality and time-varying urea. We expected urea measurements to be concentrated in the first 7 days post-randomisation. In addition, our previous work demonstrated urea changes to occur early in critical illness and therefore a priori we aimed to model associations with time-varying urea and death within the 30 day time frame [17, 21].

### Statistical analysis

We presented descriptive baseline and outcome data as medians with interquartile ranges for numerical data, and as numbers with percentages for categorical data. Raw urea trajectories are presented over time for both treatment groups. To further describe the heterogeneity in urea trajectories over time, we performed unsupervised trajectory clustering on repeated urea measurements. We used an unsupervised k-means trajectory clustering approach based on repeated log-transformed urea measurements. The number of clusters was chosen based on examination of several partition indices and our preference for selecting the smallest number of discrete clusters to illustrate trajectory variability. For a table of characteristics, patients were stratified by urea trajectory using the longitudinal clustering.

### Analysis of primary outcome

We used a joint model to quantify treatment effect of randomisation to high protein by linking the time-to-event and longitudinal outcome. The joint model can estimate two effects of protein randomisation: The direct effect of protein randomisation on survival, and the indirect effect of protein randomisation on survival through time-varying urea. The survival outcome (Cox model) and longitudinal urea measurements (linear mixed effects model) are combined in one joint model. Over time, urea is intermittently measured, has a degree of measurement error, and death stops urea measurements. Joint models allow estimation of the time-varying, patient-specific random effect of the endogenous covariate (urea) on an outcome (30-day mortality) with adjustment for baseline covariates while accounting for non-random dropouts due to death or discharge alive [23, 24].

We adapted a directed acyclic graph (DAG) to illustrate the potential causal paths tested in the joint model [25]. The elaborated, pre-specified DAG was based on expert opinion and biological plausibility, Additional file 1: Fig S1.

To build the joint model, we followed two steps. Firstly, we used a Cox model for 30-day mortality and included covariates as justified above and outlined in the DAG, Additional file 1: Fig S1. Secondly, we used a linear mixed effects model for longitudinal urea measurement. The randomisation group, measurement day, and RRT were included as fixed effects. Random intercepts and slopes were given to each subject to allow for different trajectories of urea over time. We expected urea rise to be greatest in the first 7 days in the higher protein group and introduced an interaction term for protein randomisation and time. In addition, urea was log transformed to improve likelihood of model convergence. We used cubic splines to allow for nonlinear changes of log-urea over time. The splines work by dividing the range of longitudinal urea measurements into parts that are then assigned a ‘basis function’ or ‘knots’. Three knots were chosen to capture the majority of nonlinearity based on our groups previous analyses [17, 26].

In secondary analysis, we performed several extensions of the joint models to investigate different forms of association between urea and 30-day mortality. The primary joint model estimates the association of the current value of urea at time (*t)* with the hazard of 30-day mortality, Additional file 1: Fig S2. However, patients with the same urea measurement at *t* may not have the same risk depending on the history of previous urea measurements, e.g. a patient with a decreasing urea versus a patient with an increasing urea. Therefore, we explored whether the hazard of death at time point *t* is associated with the trajectory of urea at *t* (defined by the slope incorporating the previous day's urea measurement), Additional file 1: Fig S2B. In addition, we explored the cumulative effects by examining the association of the whole area under the urea trajectory up to *t* with the time-to-event outcome, Additional file 1: Fig S2C. Model diagnostics were done by visual inspection of the diagnostic plots. The results are presented as hazard ratios (HR) with corresponding two-sided 95% credible intervals (CrIs).

### Sensitivity analysis

We performed several sensitivity analyses. Firstly, we made a joint model to adjust for baseline covariates pre-specified in the EFFORT Protein trial analysis: Age, APACHE II score, clinical frailty score, sarcopenia (SARC-F), admission type, and geographic region, where all continuous covariates were modelled as linear.

Secondly, to further test the association of urea change with protein dose and mortality, we used a multivariate joint model to include a daily POD score. This joint model tested the association of longitudinal POD score and urea with 30-day mortality. We constructed a DAG to elucidate the structure of the multivariate model, Additional file 1: Fig S3. In this multivariate analysis, we expected association of urea with 30-day mortality to remain but be smaller in magnitude than in the univariate joint model. This would suggest that the biological processes linking urea, POD, and outcome are interrelated, but that urea will continue to provide additional information on risk of death [24].

Thirdly, to address the potential confounding effect of AKI on urea rise and mortality, we repeated the primary joint model in a subgroup of patients with no AKI at baseline. Additionally, we made a multivariate joint model to include AKI status at the same time point of urea measurements (up to day 12). Acute kidney injury was defined as the presence of KDIGO stage 1 AKI or above using serum creatinine criteria [27]. This joint model tested the association of longitudinal AKI status and urea with 30-day mortality. Finally, to allow comparison with previous work linking urea-to-creatinine ratio changes to different amino acid doses, we modelled urea-to-creatinine ratio trajectories over time. [21]

### Missing data

We described the number of urea measurements over time. The joint model structure explicitly handled missingness of the longitudinal marker urea [24, 28]. In sensitivity analysis, there was missing baseline covariates and patients were excluded as specified in the main EFFORT Protein trial analysis and numbers included reported [3].

All statistical analyses were conducted using R (R Core Team, R Foundation for Statistical Computing version 4.2) and joint modelling used the *JMbayes2* package [29].

## Results

From January 17, 2018, to December 3, 2021, 1329 patients were randomised in the EFFORT Protein trial. Due to early death, discharge, or withdrawal of consent, 28 patients were excluded from the analysis. Additional exclusions were: 4 patients with missing hospital outcomes and 20 with only 1 urea measurement. 1277 patients were included in the primary analysis, Additional file 1: Fig S4. The median number of urea measurements per patient was 12 [8–12].

**Fig. 1:**
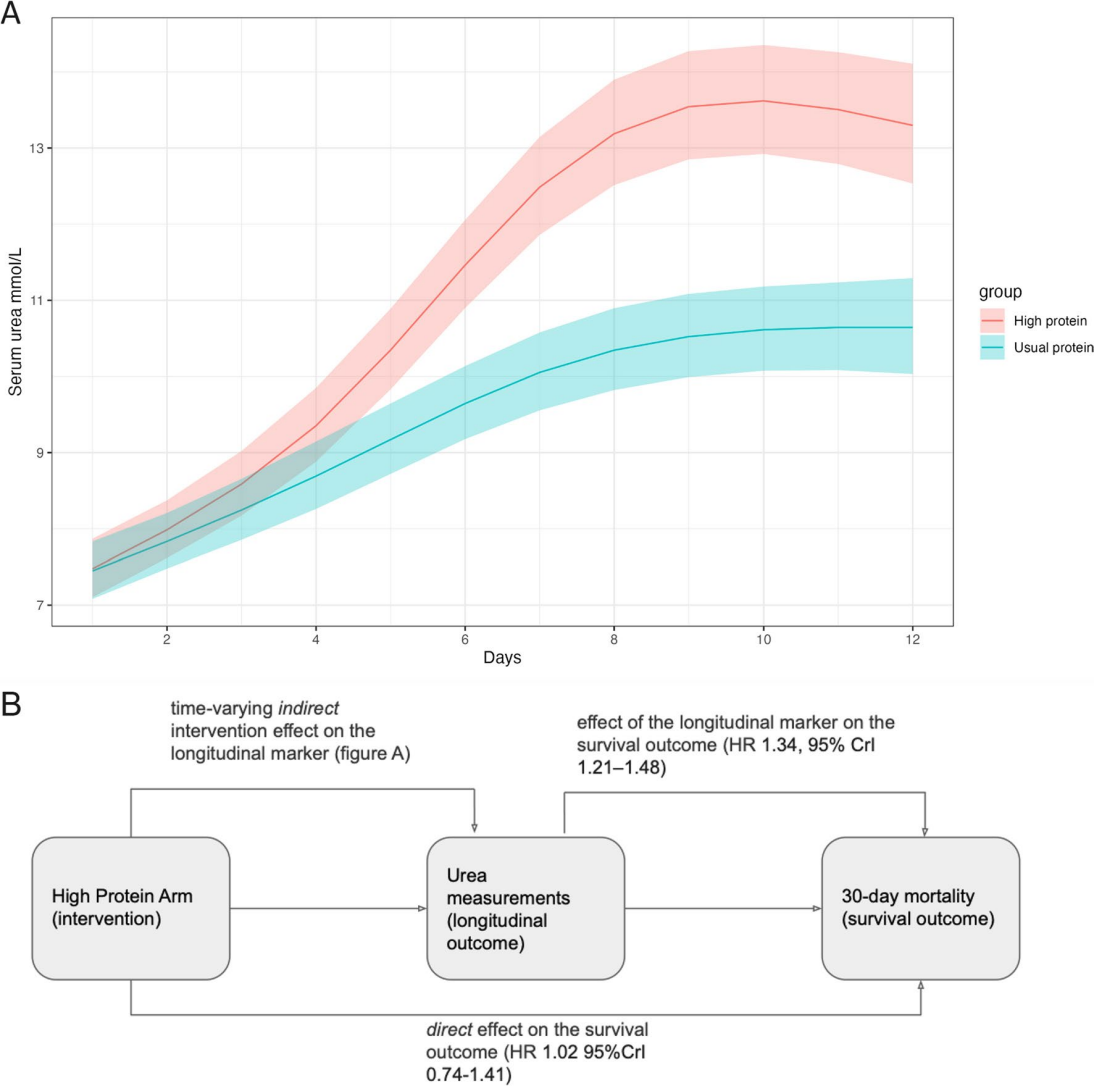
Urea trajectory for high and usual protein groups from the longitudinal sub-model (**A**), and schematic of treatment effects (**B**) from the joint model. The longitudinal sub-model (**A**) is a linear mixed effects model and includes an interaction term for time and treatment group with random intercepts and slopes. Time is modelled with a spline with three knots to account for nonlinearity of log-transformed urea values. Shown is a prediction plot with 95% prediction intervals. Predicted values are back-transformed for the plot. **B** is a schematic representation of the joint model. Time-varying indirect effect shows a strong relationship between high protein and increase in urea measurements over time. This association is combined with the effect estimate of the longitudinal marker on the survival outcome to summarise the *indirect* effect of the intervention on the survival outcome, through urea. The survival sub-model shows no *direct* effect of high protein on the survival outcome. Adapted from Oudenhoven et al.[5]

Baseline patient characteristics and primary outcomes from the EFFORT Protein trial are presented in Table 1, divided by high, medium, and low urea trajectory groups as determined by longitudinal urea measurement clusters, Additional file 1: Fig S5. In the high urea trajectory group, 23% (70/311) of patients had a diagnosis of pre-existing CKD compared to 2% (7/398) and 6% (35/568) in the low and medium groups, respectively. In the high trajectory, group 43% (133/311) received RRT on the day of randomisation compared to 3% (13/398) and 12% (70/568) in low and medium groups. Longitudinal urea profiles by randomisation group are shown in Additional file 1: Fig S6. By day 6, median urea was 2.1 mmol/L higher in the high protein group (95% CI 1.1–3.2), increasing to 3.0 mmol/L (95% CI 1.3–4.7) by day 12. Linear mixed models with splines, demonstrating increasing urea values in the high protein group as shown in Fig. 1A.

**Table 1:** Baseline characteristics by urea trajectory group

	*N*	Low	Medium	High	All patients
		(*N* = 398)	(*N* = 568)	(N = 311)	(*N* = 1277)
Age, years	1277	35/**49**/61	50/**61**/71	54/**63**/70	46/**59**/69
Female	1276	44% 175/398	40% 226/568	35% 108/310	40% 509/1276
Randomised to high protein	1277	45% 178/398	47% 269/568	60% 186/311	50% 633/1277
Admission category:	1277	75% 297/398	86% 491/568	91% 284/311	84% 1072/1277
Medical					
Surgical Elective		5% 20/398	3% 19/568	1% 4/311	3% 43/1277
Surgical Emergency		20% 81/398	10% 58/568	7% 23/311	13% 162/1277
COVID-19 positive on admission	1277	2% 8/398	10% 55/568	6% 20/311	6% 83/1277
BMI	1277	21.6/**24.8**/29.8	23.5/**26.9**/34.2	22.1/**26.4**/32.3	22.5/**26.0**/32.2
Charlson Comorbidity Index	1277	0/**0**/1	0/**0**/1	0/**1**/2	0/**0**/1
Baseline SOFA score	1277	5/ **8**/10	6/ **9**/11	8/**10**/13	6/ **9**/11
APACHE II score	1209	13.2/**18.0**/22.0	15.0/**21.0**/26.0	20.0/**25.0**/29.0	15/**21**/26
mNUTRIC score	1209	2/**4**/5	3/**5**/6	4/**6**/7	3/**5**/6
Frailty	1173	2/**3**/4	2/**3**/4	2/**3**/5	2/**3**/4
SARC-F score	1145	0/**1**/5	0/**1**/4	0/**2**/5	0/**1**/5
Renal replacement therapy on randomisation day	1277	3% 13/398	12% 70/568	43% 133/311	17% 216/1277
Acute kidney injury at time of randomisation*:	1277				
Yes		9% 35/398	22% 124/568	47% 146/311	24% 305/1277
Stage 1		46% 16/35	48% 60/124	27% 40/146	38% 116/305
Stage 2		37% 13/35	25% 31/124	18% 27/146	23% 71/305
Stage 3		17% 6/35	27% 33/124	54% 79/146	39% 118/305
Moderate or severe chronic renal disease^$^	1277	2% 7/398	6% 35/568	23% 70/311	9% 112/1277
ICU length of stay	1271	5.2/ **9.4**/17.7	5.5/**10.2**/20.2	4.9/**9.1**/18.1	5.2/**9.8**/18.5
Hospital length of stay	1269	11.0/**21.0**/38.73	9.7/**19.0**/35.9	7.3/**17.1**/38.8	9.5/**19.1**/38.0
30-day mortality	1277	0.17 67/398	0.27 153/568	0.40 124/311	0.27 344/1277
60-day mortality	1277	0.22 88/398	0.33 185/568	0.47 147/311	0.33 420/1277

Bold value indicates median

*a ****b**** c* represent the lower quartile *a*, the median *b*, and the upper quartile *c* for continuous variables. Categorical variables are described by counts and percentages. *N* is the number of non-missing values. *APACHE* acute physiology and chronic health evaluation. *ICU* intensive care unit. *SOFA* sequential organ failure assessment. *mNUTRIC* modified nutrition risk assessment in critical illness score

*Acute kidney injury refers to patients who met the criteria of KDIGO: stage 1 is at least 26.52 μmol/L increase in serum creatinine from baseline within 48 h or 1.5–1.9 times baseline within 7 days; stage 2 is 2.0–2.9 times baseline within 7 days; stage 3 is three times or more baseline within 7 days or increase to at least 353.6 μmol/L with an acute increase of more than 44.2 μmol/L

^$^Defined in comorbidities as moderate renal disease: creatinine clearance 51–85 mL/min; and severe renal disease: creatinine clearance less than 50 mL/min and not on dialysis

Trajectory groups allocated by unsupervised trajectory k-means clustering, Additional file 1: Fig S5.

There were 344 deaths at 30 days post-randomisation. Mean number of urea measurements in those that died and those that survived to day 30 were 11.0 and 11.5, respectively (*p* = 0.621, Wilcoxon rank sum test), Additional file 1: Fig S17. In the Cox model, the effect estimate for randomisation to higher protein was 1.08 (95% CI 0.88—1.34, Additional file 1: Table S1, Fig. S8, adjusted for age, baseline SOFA score, RRT on the day of randomisation, and presence of AKI). In the joint model analysis, an increase in time-varying urea was associated with an increased risk of 30-day mortality, such that a twofold increase in urea over time would be associated with a HR of 1.34, 95% CI 1.21–1.48, accounting for age, baseline SOFA score, RRT on the day of randomisation, and presence of AKI, Table 2.

**Table 2:** The estimated effect of time-varying urea and time-varying persistent organ dysfunction on 30-day ICU mortality

	Joint model of time-varying urea HR estimate (95% CrI)	Joint model of time-varying urea and persistent organ dysfunction HR estimate (95% CrI)
*Baseline variables*		
Age	1.29 (1.15–1.45)	1.30 (1.15–1.49)
SOFA	1.09 (0.97–1.23)	1.07 (0.96–1.20)
Randomisation	1.02 (0.74–1.41)	1.03 (0.76–1.41)
RRT	1.06 (0.73–1.57)	1.10 (0.78–1.56)
AKI	1.24 (0.89–1.69)	1.25 (0.91–1.69)
*Time-varying variables*		
Urea	1.34 (1.21–1.48)	1.30 (1.18–1.43)
POD	–	1.32 (1.20–1.45)

Number of subjects: 1277; number of events: 344 (26.9%); number of observations: 11,317. Joint modelling survival analysis allows estimation of the time-varying, patient-specific random effect of the endogenous covariate (urea) on an outcome. Primary and secondary joint models were adjusted for baseline variables (age, renal replacement therapy, sequential organ failure assessment, kidney dysfunction, and protein dose randomisation). Multivariate joint model was adjusted for baseline variables (age, renal replacement therapy, kidney dysfunction, and protein dose randomisation). Effect estimate is for a twofold increase in time-varying urea with 95% credible intervals. Effect estimate for the multivariate joint model is for an increase in one persistent organ dysfunction with 95% credible intervals. The hazard ratios were the adjusted hazard ratios associated with a 1-SD increment in the given variable. Values higher than 1 indicate an increased mortality. The values used for standard deviations were as follows: age, 16.7 years; and SOFA score, 3.7.

### Estimation of association between uraemia and risk of death

**Fig. 2:**
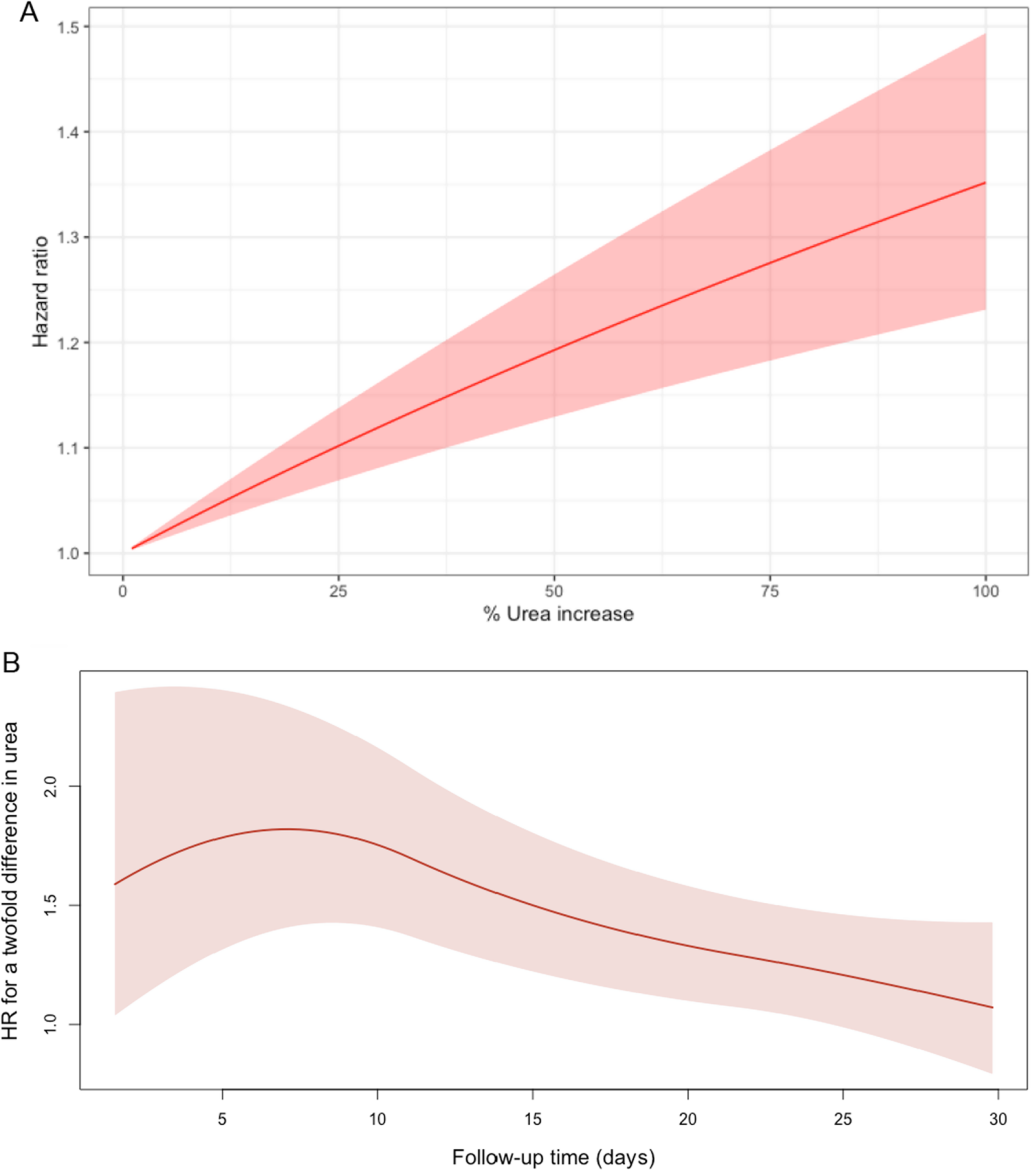
Association between time-varying urea and 30-day mortality during the EFFORT trial. Time-varying hazard ratio (HR) obtained from a Bayesian joint model estimating the association between per cent increase in time-varying urea (**A**) and 30 day mortality (*n* = 1277). **B** demonstrates the association persisted for the duration of the follow-up period. Effect on HRs illustrated by showing a twofold difference in urea at any time point during follow-up. *Shaded areas* represent 95% credible intervals

A schematic of proposed intervention effects in the joint model is shown in Fig. 1B. The time-varying *indirect* intervention effect shows a strong relationship between high protein and increase in urea over time. This association is combined with the effect estimate of the longitudinal marker on the survival outcome to summarise the *indirect* effect of the intervention on the survival outcome, through urea. For a 1% increase in time-varying urea there is a 0.4% (95% CrI 0.3–0.6%) increase in 30-day mortality, Fig. 2A. The survival sub-model suggests no *direct* effect of high protein on the survival outcome.

### Extensions of the primary joint model

Three extensions were observed. Firstly, the strength of the association between time-varying urea and 30-day mortality was persistent across the entire duration of 30-day follow-up (Fig. 2B). Secondly, increasing trajectories and higher cumulative values of urea were associated with higher risk of 30-day mortality (Additional file 1: Table S2). Whereby a doubling of the rate of rise of a patient's urea from the previous day, results in an estimated increase in the hazard of death (HR 3.42, 95% CrI 1.72–6.94). Similarly, we measured a cumulative effect of exposure to higher urea levels (area under the curve), Additional file 1: Table S2. There was an increased risk of death (HR 1.42, 95% CrI 1.22–1.65) for a doubling of the area under the curve of the urea trajectory.

### Sensitivity analyses

A second joint model adjusted for the time-varying effect of persistent organ dysfunction (POD) on the association of urea changes with mortality. Change in POD score over time is shown in Additional file 1: Fig. S9. For an increase in time-varying POD score of 1, the hazard ratio for death was 1.32 (95% CrI 1.20–1.45), Table 2. In the same model, after accounting for effect of POD, for a 1% increase in time-varying urea there was an estimated 0.37% (95% CrI 0.23–0.52) increase in 30-day mortality, Additional file 1: Fig. S10.

Time-varying urea was estimated to be associated with 30-day mortality in a joint model excluding patients with AKI at baseline (HR 1.38, 95% CrI 1.22–1.58, adjusted for; age, baseline SOFA score, and protein dose randomisation, Additional file 1: Table S3). Results were similar in a joint model excluding patients who developed AKI in the first 12 days post-randomisation (Additional file 1: Table S3). Trajectories of serum creatinine and evolution of KDIGO AKI stages over the first 12 days of ICU admission were similar between randomisation groups, Additional file 1: Fig. S11. The presence of time-varying AKI on days 1 to 12, increased the hazard of death (1.08 95%CrI 1.04–1.14, Additional file 1: Table S4). In the same model, for a 1% increase in time-varying urea there was an estimated 0.39% (95% CrI 0.29–0.54) increase in 30-day mortality, Additional file 1: Fig S12.

In a joint model adjusted for baseline covariates pre-specified in the original EFFORT Protein mortality model, an increase in time-varying urea remained associated with 30-day mortality (HR 1.31, 95% CrI 1.19–1.47, adjusted for; age, APACHE II score, clinical frailty score, sarcopenia, admission type, and geographic region, Additional file 1: Table S3). In a post hoc analysis, time-varying urea was estimated to be associated with 30-day mortality in a joint model including baseline CKD in the adjusted covariates (HR 1.34, 95% CrI 1.21–1.51, adjusted for age, baseline SOFA score, RRT on the day of randomisation, presence of AKI, chronic kidney disease, and protein dose randomisation, Additional file 1: Table S3). Urea-to-creatinine ratio increased more in patients randomised to higher protein, Additional file 1: Fig S13.

## Discussion

In this secondary analysis of the EFFORT Protein trial, higher doses of protein resulted in higher urea trajectories which were in turn associated with an increased risk of 30-day mortality. Similarly, greater rate of rise of urea and higher cumulative exposure to urea were associated with an increased risk of death. Effective randomisation to higher urea production allowed the effect of ureagenesis to be specifically examined, and that the indirect treatment effect of high-dose protein causing harm was estimated to be mediated via ureagenesis. This relationship persisted in sensitivity analyses accounting for different baseline and time-varying confounders including organ dysfunction and AKI. These data are concordant with previous similar secondary analyses of glutamine supplementation[17], and with our current understanding of skeletal muscle physiology in critical illness and specifically multi-organ dysfunction [12]. Serum urea acts as a biological signature of the metabolic derangement seen in critical illness leading to increased protein degradation.

Critically ill patients exhibit both reduced baseline muscle protein synthetic rates and an attenuated synthetic response to protein-anabolic resistance and serum urea increases reflect amino acid hepatic deamination as opposed to tissue incorporation [4, 12]. These highly energy dependent processes are limited by bioenergetic failure as a result of altered substrate utilisation [14]. Our analysis of the EFFORT study suggests that protein delivery to critically ill patients requires modification guided by the serum urea. In addition to the estimate associating time-varying urea with death, the rate of rise of urea and the cumulative exposure to greater urea levels were associated with death. These consistent associations support the potential benefit of urea guided strategies. Firstly, to avoid a protein dose initially that amplifies the rate of rise of urea in early organ failure. Secondly, to limit the cumulative exposure to high urea levels from protein dosing. Whether this approach improves patient outcomes requires prospective testing. However, such a strategy represents a promising avenue to move the critical care rehabilitation field forward in terms of patient selection and efficacy signals.

### Ureagenesis

While endogenous and exogenous amino acids can be used for ATP generation in starvation (e.g. via the Cahill cycle) this is not necessarily true in multi-organ dysfunction. Intramuscular hypoxia and inflammatory signalling, impaired GLUT-4 translocation and insulin resistance either divert key processes or provide negative feedback loops to prevent this [10–12]. The end result is an accumulation of toxic intermediary metabolites such as ammonia from an overburdened urea cycle. Multi-organ dysfunction can include subtle liver dysfunction, which may affect such pathways in the absence of overt hepatic failure. Quantifying the effect of hepatic dysfunction on urea cycle flux (and indeed Cahill cycle flux) was outside the scope of the reanalysis, though the retention of the signal for harm following correction for persistent organ dysfunction implies that this may have been accounted for. In previous work, ourselves and others have demonstrated the relationship between mortality, morbidity, and persistent critical illness with the urea-to-creatinine ratio [21]. Urea-to-creatinine ratio increased in patients randomised to higher protein suggesting an increase in catabolism. However, loss of creatinine generation related to muscle wasting was not assessed in this reanalysis, as the focus was on the much closer relationship between protein dose and urea metabolism.

Although AKI is an independent cause of uraemia, the fact that randomisation to higher protein was associated with higher urea and that harm associated with high-dose protein was associated with presence of AKI suggests that the two causes of uraemia (decreased elimination and increased production) may potentiate in their associations with adverse outcome. By contrast, clinical need to commence RRT is triggered by a supply demand imbalance with respect to renal function and metabolic burden and thus also reflects both excess urea generation and inadequate elimination. In this study, effective randomisation to higher urea production allows the effect of ureagenesis to be specifically examined. Our analyses suggest considerations for protein loading in critical illness may equally apply to patients with AKI, with or without RRT.

### Clinical implications and future directions

The findings of a biological basis for the potential for harm from high protein doses in the critically ill patient strengthens the case for not exceeding the current guidance doses of 1.2 g/kg at any stage in critical illness, especially in the setting of a raised or rising urea. Multiple RCTs have demonstrated increases in urea with varying doses of amino acids or enhanced feeding [30]. Clinical guidelines may be revised to remove higher doses, or to consider serum urea prior to dose escalation. More specifically, time-varying urea could be assessed post initiation of nutrition, to account for baseline heterogeneity. While the nuances of clinical implementation of such methods require elucidating, clinicians should consider modifying protein delivery to critically ill patients when a rising urea is observed following protein loading. Future research might focus on urea cycle activity and the mechanisms of tissue ammonia toxicity, and indeed if this is the mechanism of the altered cellular processes characterising multi-organ dysfunction in critical illness. In addition to better characterisation of nitrogen excretion in multi-organ dysfunction, these effects need to be considered alongside other potential mechanisms of harm of amino acids such as altered autophagy [31]. Potential avenues include large dataset analyses with functional outcome measures, observational studies examining dose-dependent ammonia toxicity, or mediation-based examination of clinical trials.

### Strengths and limitations

These analyses remain secondary to the main analysis of the EFFORT Protein trial and have several limitations. Our estimate of the potential causal relationship between time-varying urea and 30-day mortality remains dependent on the assumption that appropriate confounders have been accounted for. This strong assumption suggests residual baseline and time-varying confounding has been addressed and the shared interdependencies between the longitudinal urea measurements and 30-day mortality are explained by latent, subject-specific random effects [23]. The persistence of the association between urea rise and mortality in joint models with a variety of association structures and in models incorporating time-varying organ dysfunction, or specifically AKI status, adds support to a putative causal relationship. In sensitivity analyses, we were able to test the association using the original EFFORT Protein adjustment set to provide consistency with pre-selected, clinically relevant confounders. Finally, when we removed any effect of baseline AKI, an important subgroup with a signal for harm in the EFFORT Protein, urea increases remained associated with mortality. These analyses add important evidence to support urea as a surrogate on the causal pathway in proteins’ effect on mortality [7].

A second limitation is that these analyses were not what the EFFORT Protein trial was designed for and remain at risk of residual bias. The joint model approach and adjustment sets were not pre-specified and there remains risk of confounding, especially from additional confounding introduced by examining a relationship with a surrogate (urea) over time [32]. However, when interest is to gain understanding into the process of how a treatment affects the survival outcome via a biomarker pathway, joint models provide an attractive framework and can potentially distinguish both the indirect treatment effect on the survival outcome through the longitudinal outcome, and the direct treatment effect on the survival outcome [9]. Third, as a pragmatic multicentre RCT, there are no mechanistic data from the EFFORT Protein trial to elucidate the biological pathway involved between excess ureagenesis, organ damage, and mortality. Consequently, we can only provide a conceptual framework regarding potential mechanisms of harm which may involve ammonia. In addition, the multitude of potential pathways that can impact catabolism such as inflammation and immobilisation[33] are not directly measured and therefore limit the assessment of urea as a marker of nutritional resistance. However, when persistent organ dysfunction and need for ongoing organ support—broad but relatively robust surrogate endpoints of the pathophysiology of critical illness—were included in joint models, urea rises remained associated with harm. Fourth, we do not have detailed data on incidence of gastrointestinal bleeding which could have increased urea and confounded these analyses. Finally, EFFORT Proteins, pragmatic RCT design provides robust average treatment effects (such as higher protein causing higher urea levels), but how generalisable these effects are on an individual patient level with differing baseline risk remains unknown.

## Conclusions

The increased risk of death in sicker patients randomised to a higher protein dose in the EFFORT Protein trial was estimated to be mediated by increased urea cycle activity, of which serum urea is a biological signature. Serum urea should be taken into consideration when initiating and continuing protein delivery in critically ill patients.

### Supplementary Information

**Additional file 1.** Online Data Supplement.

## Data Availability

Data collected for the underlying EFFORT protein study will not be publicly available but are being used internally for secondary purposes. Data dictionary or other study tools are available from the coordinating authors (DKH and AD) upon request.

## References

[1] Compher C, Bingham AL, McCall M (2022). Guidelines for the provision of nutrition support therapy in the adult critically ill patient: The American Society for Parenteral and Enteral Nutrition. J Parenter Enter Nutr.

[2] Patel JJ, Rice T, Compher C, Heyland DK (2020). Do we have clinical equipoise (or uncertainty) about how much protein to provide to critically Ill patients?. Nutr Clin Pract.

[3] Heyland DK, Patel J, Compher C (2023). The effect of higher protein dosing in critically ill patients with high nutritional risk (EFFORT Protein): an international, multicentre, pragmatic, registry-based randomised trial. The Lancet.

[4] Puthucheary ZA, Rawal J, McPhail M (2013). Acute skeletal muscle wasting in critical illness. JAMA.

[5] van Oudenhoven FM, Swinkels SHN, Hartmann T, Rizopoulos D (2022). Modeling the underlying biological processes in Alzheimer’s disease using a multivariate competing risk joint model. Stat Med.

[6] Reddy K, Sinha P, O’Kane CM (2020). Subphenotypes in critical care: translation into clinical practice. Lancet Respir Med.

[7] Harhay MO, Casey JD, Clement M (2020). Contemporary strategies to improve clinical trial design for critical care research: insights from the First Critical Care Clinical Trialists Workshop. Intensive Care Med.

[8] van Eijk RPA, Roes KCB, van den Berg LH, Lu Y (2022). Joint modeling of endpoints can be used to answer various research questions in randomized clinical trials. J Clin Epidemiol.

[9] van Oudenhoven FM, Swinkels SHN, Ibrahim JG, Rizopoulos D (2020). A marginal estimate for the overall treatment effect on a survival outcome within the joint modeling framework. Stat Med.

[10] Heyland D, Muscedere J, Wischmeyer PE (2013). A randomized trial of glutamine and antioxidants in critically ill patients. N Engl J Med.

[11] Heyland DK, Wibbenmeyer L, Pollack JA (2022). A randomized trial of enteral glutamine for treatment of burn injuries. N Engl J Med.

[12] Chapple LS, Kouw IWK, Summers MJ (2022). Muscle protein synthesis after protein administration in critical illness. Am J Respir Crit Care Med.

[13] Taguchi A, Fahrmann JF, Hanash SM (2020). A promising CPS1 inhibitor keeping ammonia from Fueling cancer. Cell Chem Biol.

[14] Puthucheary ZA, Astin R, Mcphail MJW (2018). Metabolic phenotype of skeletal muscle in early critical illness. Thorax.

[15] Vilstrup H, Amodio P, Bajaj J (2014). Hepatic encephalopathy in chronic liver disease: 2014 practice guideline by the american association for the study of liver diseases and the European association for the study of the liver. Hepatol Baltim Md.

[16] Bajaj JS, Bloom PP, Chung RT (2020). Variability and lability of ammonia levels in healthy volunteers and patients with cirrhosis: implications for trial design and clinical practice. Am J Gastroenterol.

[17] Haines RW, Fowler AJ, Wan YI (2022). Catabolism in critical illness: a reanalysis of the reducing deaths due to oxidative stress (REDOXS) trial*. Crit Care Med.

[18] Heyland DK, Patel J, Bear D (2019). The effect of higher protein dosing in critically Ill patients: a multicenter registry-based randomized trial: the EFFORT trial. J Parenter Enter Nutr.

[19] Breen L, Phillips SM (2011). Skeletal muscle protein metabolism in the elderly: interventions to counteract the “anabolic resistance” of ageing. Nutr Metab.

[20] Zhu R, Allingstrup MJ, Perner A (2018). The effect of IV amino acid supplementation on mortality in ICU patients may be dependent on kidney function: post hoc subgroup analyses of a multicenter randomized trial. Crit Care Med.

[21] Haines RW, Zolfaghari P, Wan Y (2019). Elevated urea-to-creatinine ratio provides a biochemical signature of muscle catabolism and persistent critical illness after major trauma. Intensive Care Med.

[22] Heyland DK, Elke G, Cook D (2015). Glutamine and antioxidants in the critically ill patient: a post hoc analysis of a large-scale randomized trial. JPEN J Parenter Enteral Nutr.

[23] Urner M, Jüni P, Hansen B (2020). Time-varying intensity of mechanical ventilation and mortality in patients with acute respiratory failure: a registry-based, prospective cohort study. Lancet Respir Med.

[24] Rizopoulos D, Ghosh P (2011). A Bayesian semiparametric multivariate joint model for multiple longitudinal outcomes and a time-to-event. Stat Med.

[25] Ibrahim JG, Chu H, Chen LM (2010). Basic concepts and methods for joint models of longitudinal and survival data. J Clin Oncol.

[26] Harrell FE (2001). Regression Modeling Strategies: With Applications to Linear Models, Logistic Regression, and Survival Analysis.

[27] Group K (2012). KDIGO clinical practice guideline for acute kidney injury. Kidney Int Suppl.

[28] Joint Models for Longitudinal and Time-to-Event Data: With Applications in R. In: Routledge CRC Press. https://www.routledge.com/Joint-Models-for-Longitudinal-and-Time-to-Event-Data-With-Applications/Rizopoulos/p/book/9781439872864. Accessed 31 Jul 2022

[29] Rizopoulos D (2022) JMbayes2: Extended Joint Models for Longitudinal and Time-to-Event Data

[30] Gunst J, Casaer MP, Preiser J-C (2023). Toward nutrition improving outcome of critically ill patients: How to interpret recent feeding RCTs?. Crit Care.

[31] Hermans G, Casaer MP, Clerckx B (2013). Effect of tolerating macronutrient deficit on the development of intensive-care unit acquired weakness: a subanalysis of the EPaNIC trial. Lancet Respir Med.

[32] Mansournia MA, Etminan M, Danaei G (2017). Handling time varying confounding in observational research. BMJ.

[33] Vanhorebeek I, Latronico N, Van den Berghe G (2020). ICU-acquired weakness. Intensive Care Med.

