# Supplementary material for: Association between urea trajectory and protein dose in critically ill adults: a secondary exploratory analysis of the effort protein trial (RE-EFFORT)
Source: Crit Care. 2024 Jan 16;28:24. doi: 10.1186/s13054-024-04799-1 (PMC10792897; doi:10.1186/s13054-024-04799-1)

Online Data Supplement

Association Between Urea and Protein Dose in Critical Illness: Exploratory Analysis of the EFFORT Protein Trial

Ryan W. Haines, John R. Prowle, Andrew Day, Danielle E. Bear, Daren K. Heyland, Zudin Puthucheary

Online Data Supplement

This supplement has additional information on methods and results, organised as:

1. **Covariates and joint model construction**
2. **Consort and data missingness**
3. **Additional analyses**

1. Covariates and joint model construction

Please, refer to the figures within the next pages.

- **Figure E1:** Directed acyclic graph for primary joint model.
- **Figure E2:** Different joint model structures
- **Figure E3:** Illustrative directed acyclic for the multivariate joint model

Figure E1. Directed acyclic graph for primary joint model. The green node represents exposure (high protein randomisation). Urea trajectory and 30-day mortality are outcomes and other blue node potential confounders. Arrows demonstrate potential causal paths, e.g. Acute Kidney Injury (AKI) can result in need for Kidney Replacement therapy (KRT) and an increase in 30-day mortality.

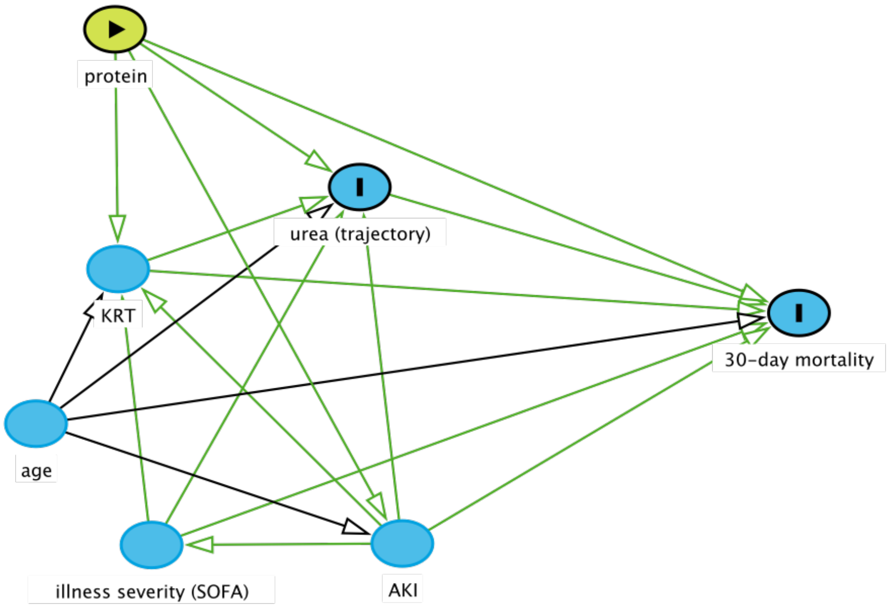

Baseline covariates age; increasing age is associated with increased illness severity and potential increase in inability to process excess protein load. Illness severity (SOFA score at baseline); increased severity of illness may affect the capability to process excess protein, with more severely ill patients then experiencing the negative effects of mal-processed amino acids. Acute kidney injury (at enrolment); Kidney function and kidney support may affect the metabolic impact of higher protein doses. Some observational data has suggested harm from amino acid delivery in patients with AKI. Renal replacement therapy (RRT; administered on the day of enrolment). There is an increased risk of mortality in patients receiving RRT while extra-corporeal clearance of urea alters longitudinal urea trajectory.

```
dag {
bb="-6.036,-6.97,3.985,5.322"
"30-day mortality" [outcome,pos="3.012,-1.759"]
"illness severity" [pos="-2.371,1.130"]
"urea (trajectory)" [outcome,pos="-0.640,-3.326"]
AKI [pos="-0.286,1.111"]
KRT [pos="-2.649,-2.306"]
age [pos="-3.332,-0.380"]
protein [exposure,pos="-2.675,-5.290"]
"illness severity" -> "30-day mortality"
"illness severity" -> "urea (trajectory)"
"illness severity" -> KRT
"urea (trajectory)" -> "30-day mortality"
AKI -> "30-day mortality"
AKI -> "illness severity"
AKI -> "urea (trajectory)"
AKI -> KRT
KRT -> "30-day mortality"
KRT -> "urea (trajectory)"
age -> "30-day mortality"
age -> "urea (trajectory)"
age -> AKI
age -> KRT
```

```
protein -> "30-day mortality"
protein -> "urea (trajectory)"
protein -> AKI
protein -> KRT
}
```

Figure E2. Graphical representation different association structure between the longitudinal (repeated urea measurements) and survival (30-day mortality) outcomes. (A) shows the current value, (B) the rate of change, or slope, and (C) the cumulative effect (area under the curve) of the longitudinal trajectory. Adapted from Oudenhoven.(1)

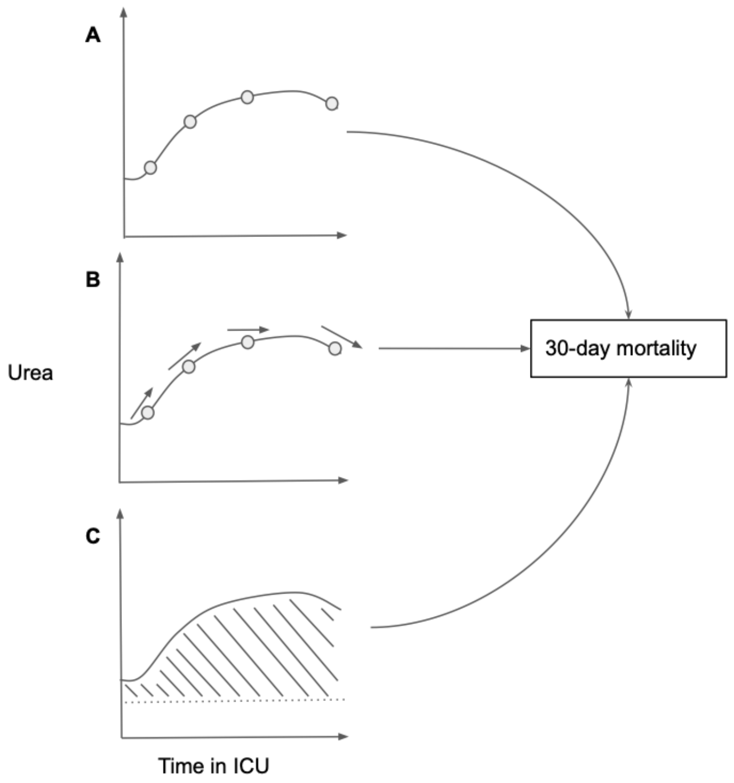

Figure E3. Illustrative directed acyclic graph describing the structure of the multivariate joint model estimating treatment effect of higher protein dose on time-varying urea, time-varying persistent organ dysfunction score, and time-to-event outcome e.g 30-day mortality. *t0* is baseline measurement and *ti* represents repeated measurements (*t1,2…i*).

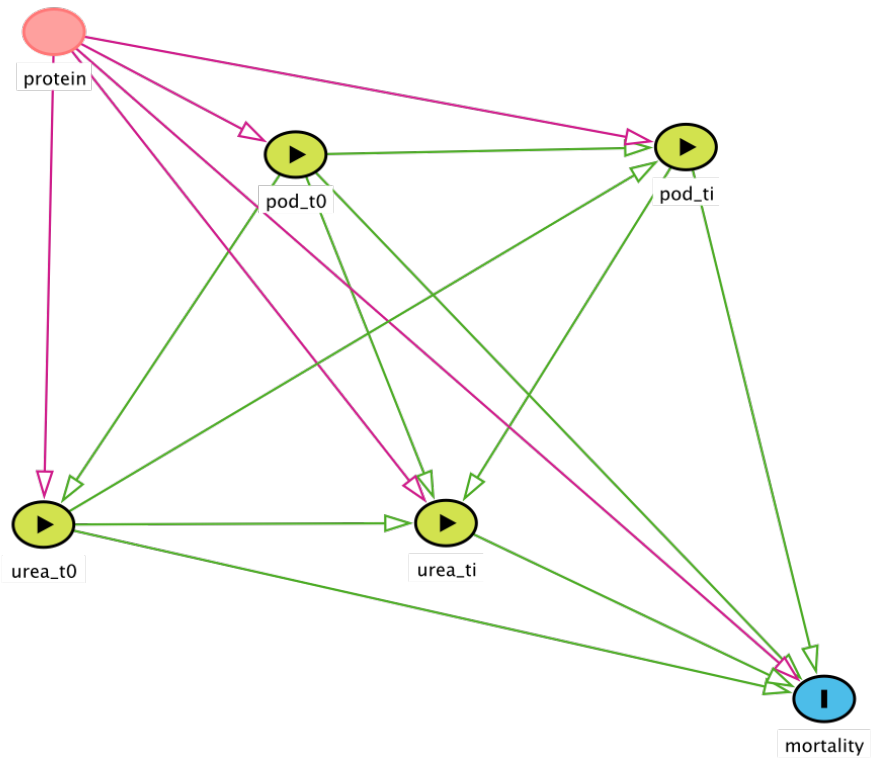


```
dag {
bb="-6.036,-6.97,3.985,5.322"
mortality [outcome,pos="2.620,1.980"]
pod_t0 [exposure,pos="-1.777,-4.799"]
pod_ti [exposure,pos="1.470,-4.893"]
protein [pos="-3.787,-6.328"]
urea_t0 [exposure,pos="-3.875,-0.191"]
urea_ti [exposure,pos="-0.526,-0.210"]
pod_t0 -> mortality
pod_t0 -> pod_ti
pod_t0 -> urea_t0
pod_t0 -> urea_ti
pod_ti -> mortality
pod_ti -> urea_ti
protein -> mortality
protein -> pod_t0
protein -> pod_ti
protein -> urea_t0
protein -> urea_ti
urea_t0 -> mortality
urea_t0 -> pod_ti
urea_t0 -> urea_ti
urea_ti -> mortality
}
```

2. Consort and data missingness

Please, refer to the figures within the next pages.

- **Figure E4:** Consort diagram
- **Figure E5:** Longitudinal urea trajectory clustering
- **Figure E6:** Longitudinal urea trajectory clustering Longitudinal urea trajectories
- **Figure E7:** Summary of urea measurements

Figure E4. Consort diagram

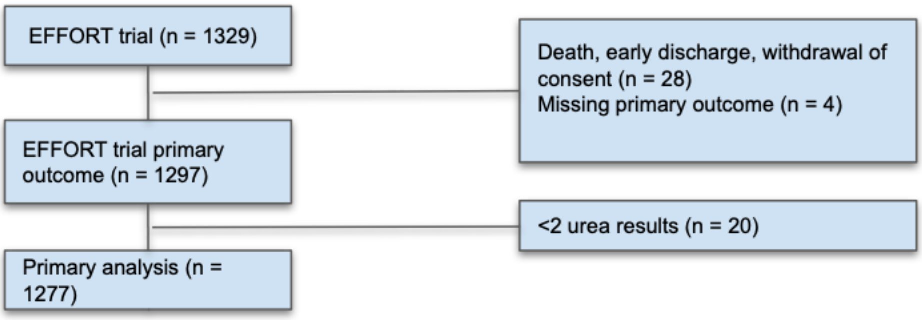

Figure E5. Longitudinal k-means clustering analysis trajectory clusters. Using an unsupervised machine learning technique, we performed k-means trajectory clustering based on repeated urea measurements using the *kml* package. We used a Euclidean distance with Gower adjustment for clustering of trajectories. Partitioning of trajectories is visualised in real time allowing inspection of clustering patterns with additional number of clusters added. The *kml* package uses standardised criterions for cluster number selection including: Calinski-Harabasz, Ray-Turi index and, Davies-Bouldin index. For missing data, the *kml* package uses linear interpolation. Three clusters were chosen based on maximisation of these indices and patient numbers within each cluster.

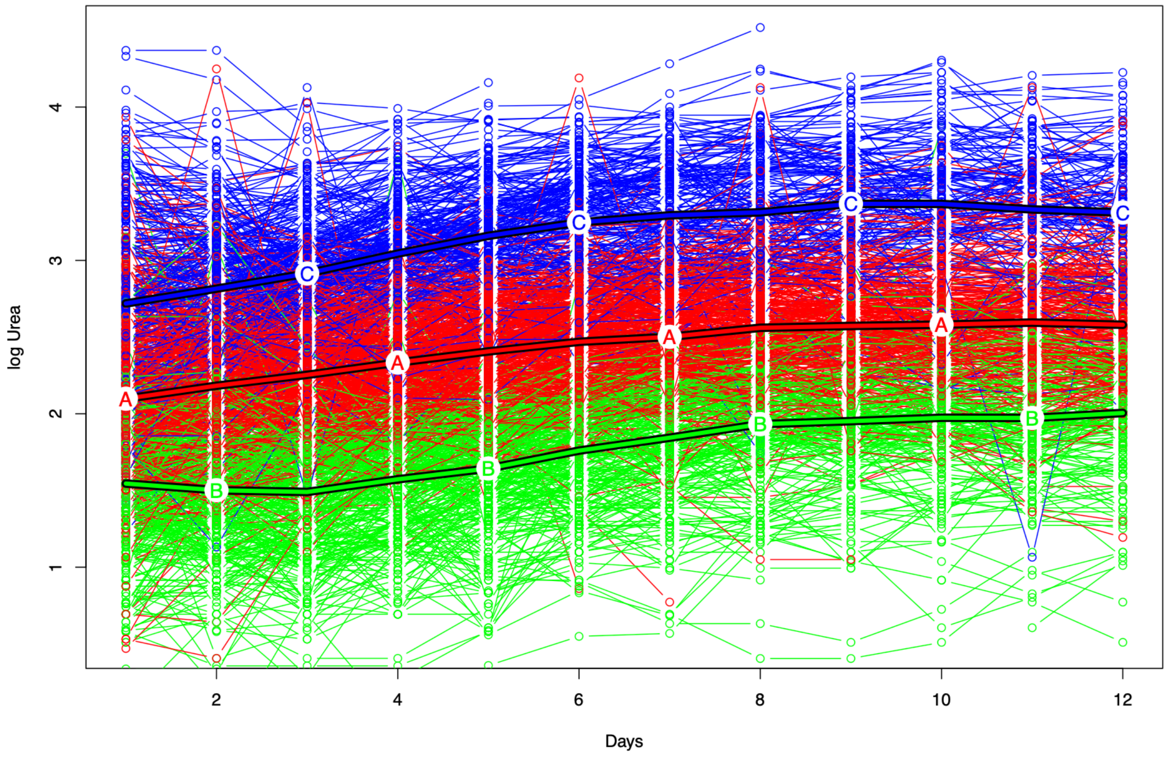

Figure E6. Longitudinal urea trajectories by randomised protein treatment group. *A is a snapshot or an interactive plot created using dReport function in R package hReport with medians (dot) and quantile intervals (intervals from shortest to longest lines are 0.375 – 0.625, 0.250 – 0.750, 0.125 – 0.875, and 0.050 – 0.950).*

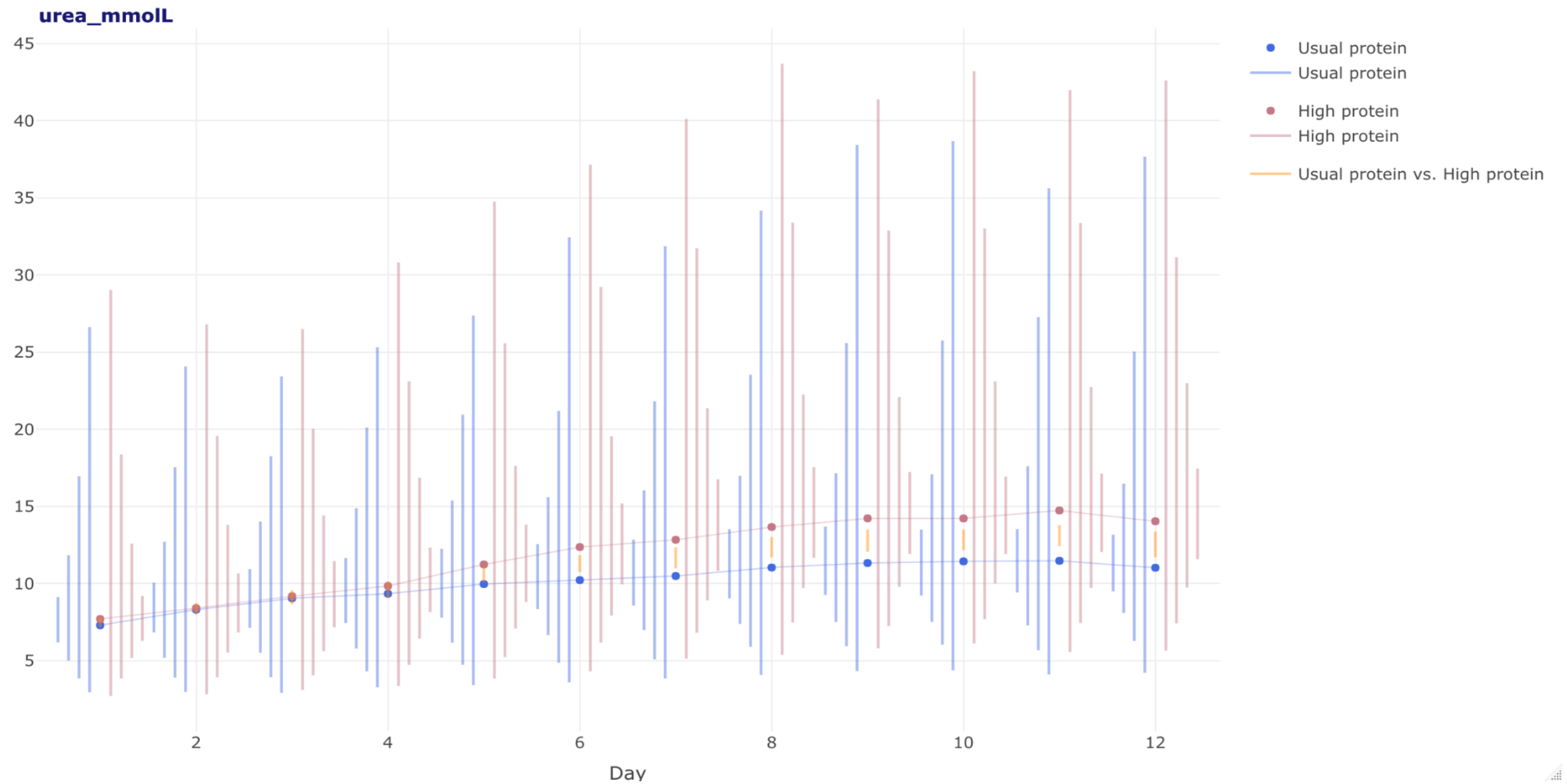

Figure E7. Box plot of number of urea measurements comparing patients that died and survived. Green lines are 25th and 75th percentiles. Blue dots are mean. *Plot created using summaryM function in R package Hmisc.*

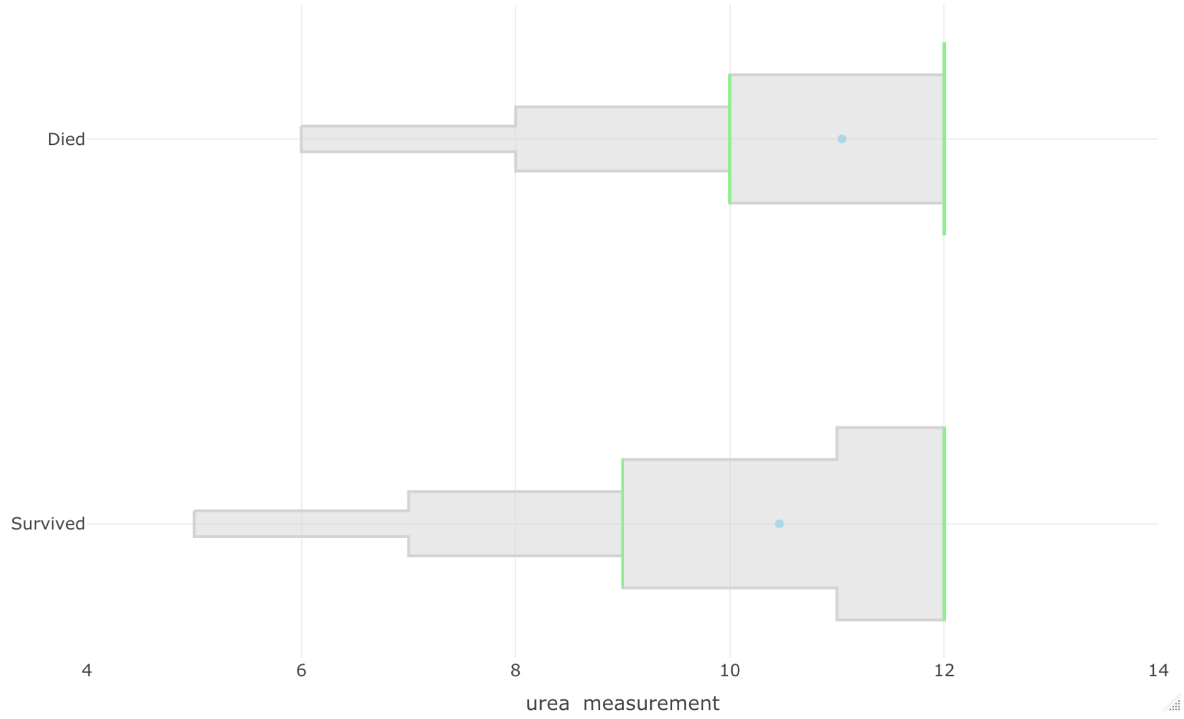

3. Additional analyses

Please, refer to the tables and figures within the next pages.

- **Figure E8.** Cox model for 30-day mortality
- **Table E1.** Cox model for 30-day mortality
- **Table E2.** Joint models for urea rise (slope) and area under the curve of the urea trajectory (cumulative)
- **Figure E9.** Alluvial plot of change in organ failures over time
- **Figure E10.** Association between time-varying urea and 30-day mortality using a multivariate joint model including time-varying persistent organ dysfunction score.
- **Table E3.** Joint model sensitivity analysis
- **Figure E11.** Longitudinal creatinine trajectories (A) and change in acute kidney injury stage over time (B).
- **Table E4.** Multivariate joint model for time-varying urea, time-varying acute kidney injury and 30-day ICU mortality.
- **Figure E12.** Association between time-varying urea and 30-day mortality using a multivariate joint model including time-varying acute kidney injury.
- **Figure E13.** Longitudinal urea-to-creatinine ratio trajectories.

Figure E8. (A) Cox model for 30-day mortality adjusted for age, baseline SOFA score, KRT on day of randomisation, and presence of AKI at randomisation. 90, 95, and 99% confidence intervals shown in shades of blue. 75th vs 25th centile comparisons for continuous variables. **(B)** Prediction plot, showing the relationship between each predictor and the log relative hazard. *SOFA*, sequential organ failure assessment; *KRT*, kidney replacement therapy; *AKI*, acute kidney injury. *Plots created using R package rms*.

A

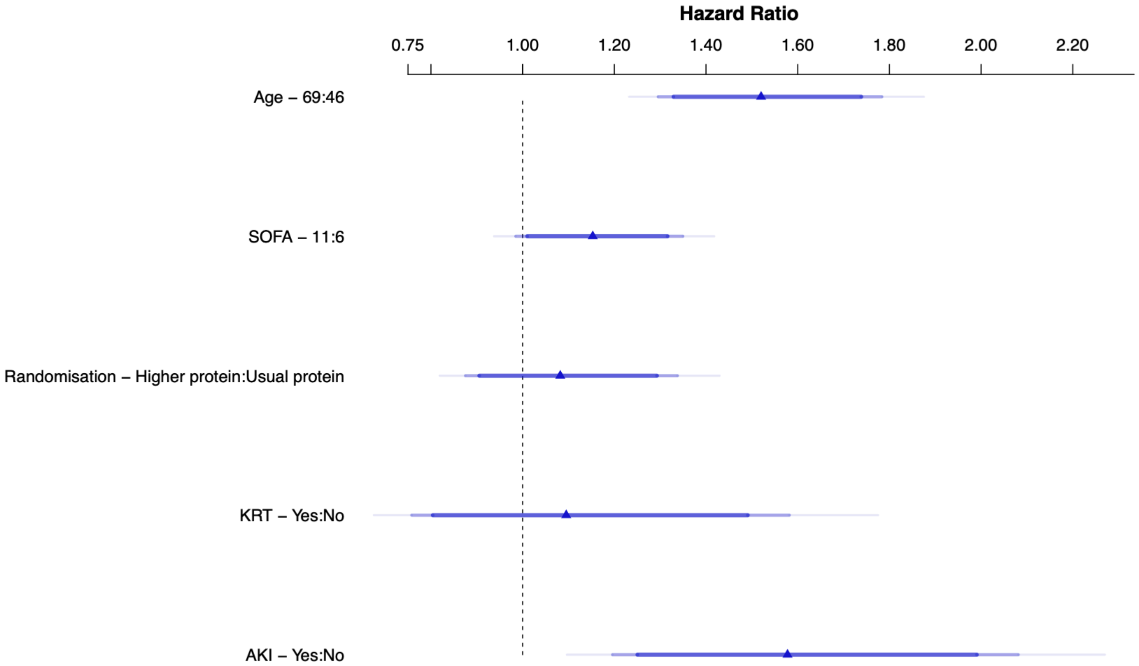

B

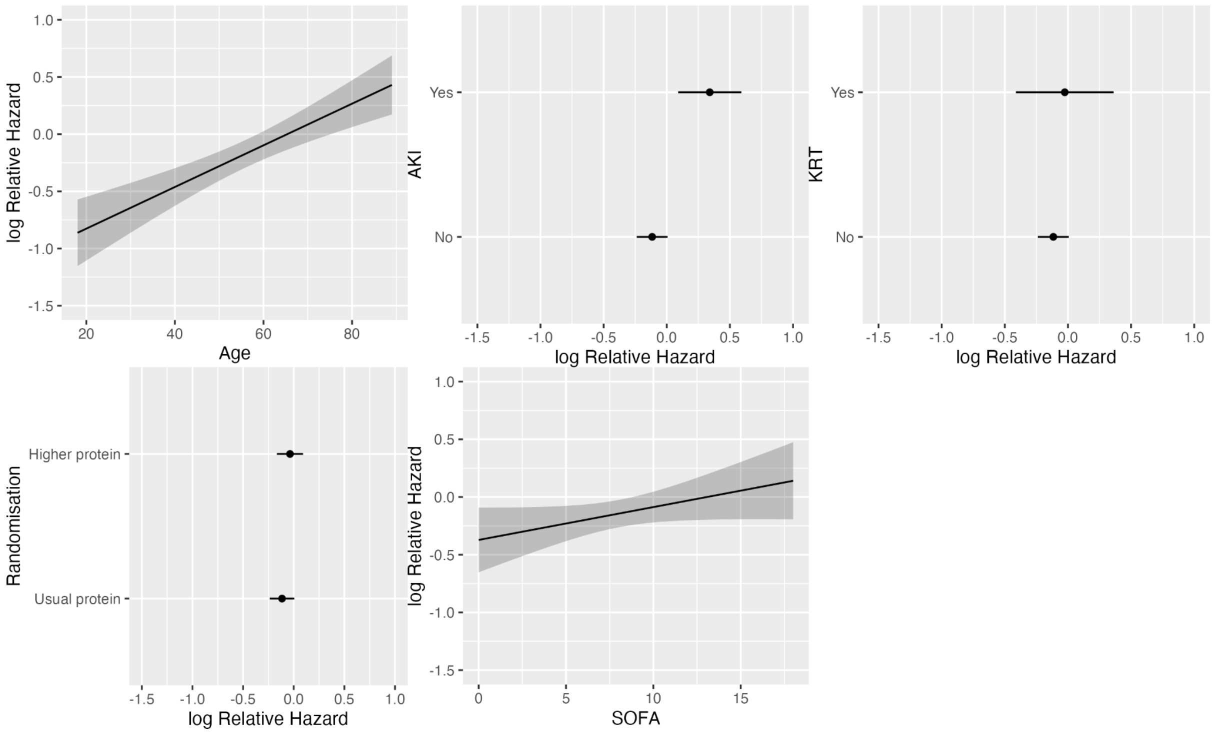

Table E1. Cox model for 30-day mortality adjusted for age, baseline SOFA score, KRT in the first 24 hours of randomisation, and presence of AKI at randomisation.

	**Low**	**High**	**Difference**	**Effect**	**S.E.**	**Lower 0.95**	**Upper 0.95**
Age	46	69	23	0.419	0.08147	0.259	0.579
Hazard Ratio	46	69	23	1.520		1.296	1.783
SOFA	6	11	5	0.142	0.08026	-0.0150	0.230
Hazard Ratio	6	11	5	1.153		0.985	1.349
Randomisation - Higher Protein: Usual Protein	1	2		0.079	0.10810	-0.133	0.291
Hazard Ratio	1	2		1.082		0.876	1.337
KRT - Yes:No	1	2		0.091	0.18750	-0.277	0.458
Hazard Ratio	1	2		1.095		0.758	1.581
AKI - Yes:No	1	2		0.456	0.14120	0.179	0.733
Hazard Ratio	1	2		1.578		1.196	2.081

Table E2. The estimated effect of time-varying urea on 30-day ICU mortality using different types of joint model.

	**Joint model of time-varying urea - slope** HR estimate (95% CrI)	**Joint model of time-varying urea - cumulative** HR estimate (95% CrI)
**Baseline variables**		
Age	1.37 (1.22-1.55)	1.28 (1.13-1.46)
SOFA	1.13 (1.01-1.26)	1.07 (0.96-1.20)
Randomisation	1.06 (0.77-1.46)	1.02 (0.74-1.41)
RRT	1.63 (1.17-2.23)	1.31 (0.92-1.69)
AKI	1.65 (1.23-2.17)	1.26 (0.93-1.67)
**Time-varying variables**		
Urea	3.42 (1.72–6.94)	1.42 (1.22–1.65)

Number of subjects: 1277; Number of events: 344 (26.9%); Number of observations: 11317. Joint modelling survival analysis allows estimation of the time-varying, patient-specific random effect of the endogenous covariate (urea) on an outcome. Joint models were adjusted for baseline variables (age, renal replacement therapy, sequential organ failure assessment, acute kidney injury, and protein dose randomisation). Effect estimate is for a two-fold increase in rate of urea rise (slope) and area under the curve of the urea trajectory (cumulative) with 95% credible intervals. The hazard ratios were the adjusted hazard ratios associated with a 1-SD increment in the given variable. Values higher than 1 indicate an increased mortality. The values used for standard deviations were as follows: Age, 16.7 years; and SOFA score, 3.7.

Figure E9. Alluvial plot of persistent organ dysfunction score over 30-day trial period. Persistent organ dysfunction was scored daily for mechanical ventilation, vasopressor therapy, and kidney replacement therapy.

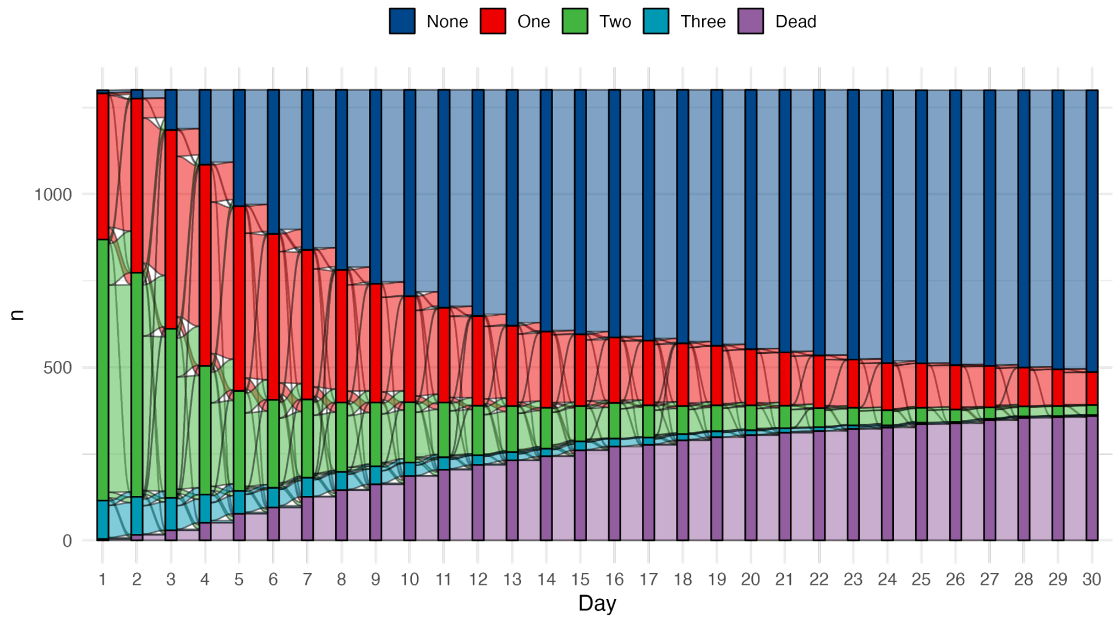

Mechanical ventilation: if any part of the calendar day is on or between the start and stop date of any invasive mechanical ventilation period, or the patient restarted invasive mechanical ventilation within 48 hours of the current day, then then the day is not a free day.

Vasopressor therapy: days with more than 2 hours of any dose of norepinephrine, epinephrine, vasopressin, dobutamine, milrinone or levosimendan and >5 ug/kg/min of dopamine, or > 50 ug/minute of phenylephrine, will not be considered free days. The 48-hour rule does not apply to vasopressor therapy or renal replacement therapy.

Renal replacement therapy: if any part of the calendar of the calendar day is on or between the start and stop date of any renal replacement therapy period then the day is not a free day.

Figure E10. Association between time-varying urea and 30-day mortality using a multivariate joint model including time-varying persistent organ dysfunction score.

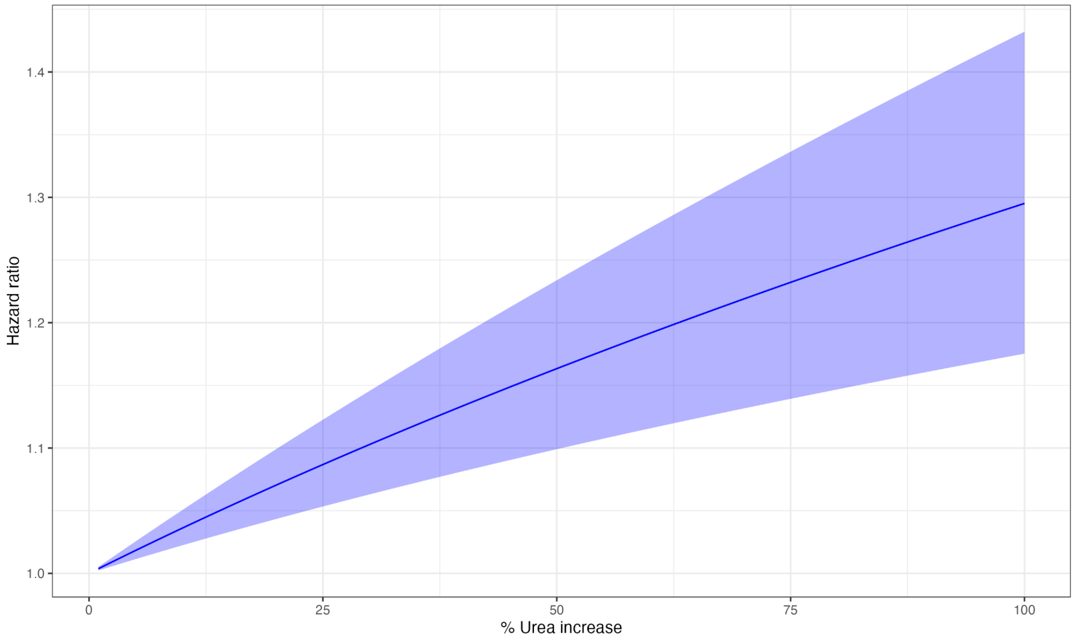

Table E3. The estimated effect of time-varying urea on 30-day ICU mortality in i) excluding patients with AKI at baseline, ii) excluding patients with AKI in the first 12 days, iii) adjusted for pre-specified EFFORT covariates, and iv) adjusted for chronic kidney disease at baseline.

	**Time-varying urea**<br>HR estimate (95% CrI)
**i) Joint model of time-varying urea - AKI removed**<br>N = 952	1.38 (1.22 - 1.58)
**i) Joint model of time-varying urea – AKI in first 12 days removed**<br>N = 787	1.34 (1.14 – 1.58)
**iii) Joint model of time-varying urea - EFFORT adjusted**<br>N = 1077	1.31 (1.19 - 1.47)
**iv) Joint model of time-varying urea - CKD adjusted**<br>N = 1277	1.34 (1.21 - 1.51)

Joint modelling survival analysis allows estimation of the time-varying, patient-specific random effect of the endogenous covariate (urea) on an outcome. Joint model i) was adjusted for baseline variables (age, sequential organ failure assessment, and protein dose randomisation) and excluded patients with acute kidney injury on the day of randomisation. Joint model ii) was adjusted for baseline variables (age, sequential organ failure assessment, and protein dose randomisation) and excluded patients with acute kidney injury occurring in the first 12 days post randomisation. Joint model iii) was adjusted for covariates pre-specified in the EFFORT trial including: age, APACHE II score, clinical frailty score, sarcopenia, admission type and geographic region. 200 patients excluded for missing baseline covariates. Joint model iv) was adjusted for age, baseline SOFA score, RRT on the day of randomisation, presence of AKI, chronic kidney disease, and protein dose randomisation. Effect estimate is for a two-fold increase in time-varying urea with 95% credible intervals. Values higher than 1 indicate an increased mortality.

Figure E11. Longitudinal creatinine trajectories (A) and change in acute kidney injury stage over time (B).

A

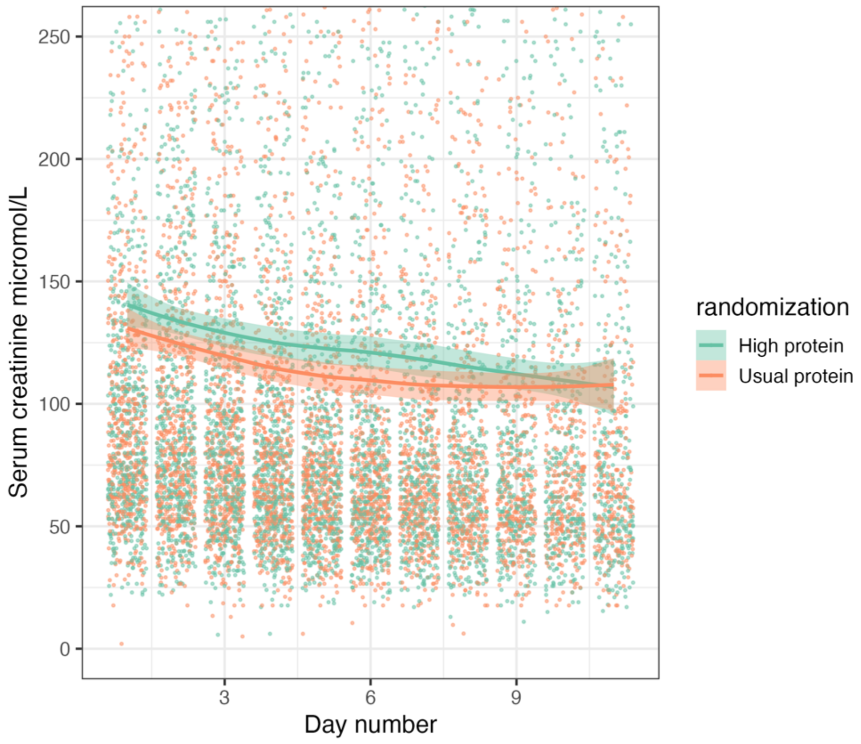

B

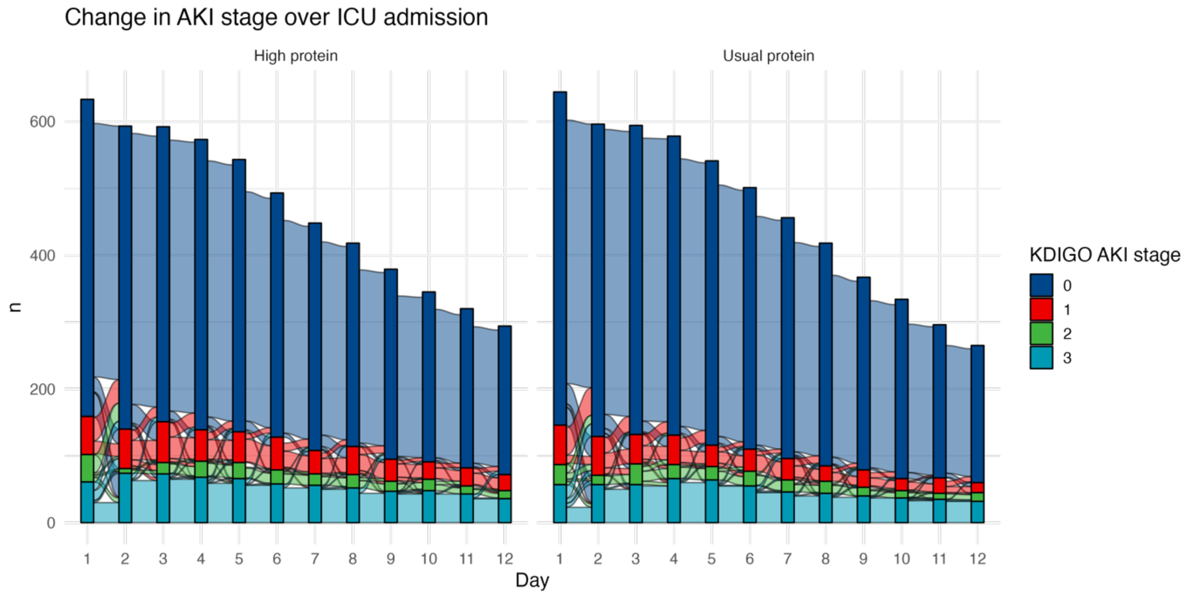

Table E4. The estimated effect of time-varying urea and time-varying acute kidney injury on 30-day ICU mortality.

	**Joint model of time-varying urea and acute kidney injury** HR estimate (95% CrI)
**Baseline variables**	
Age	1.28 (1.12-1.46)
SOFA	1.12 (1.00-1.26)
Randomisation	1.01 (0.74-1.42)
RRT	1.07 (0.75-1.51)
**Time-varying variables**	
Urea	1.31 (1.19-1.73)
AKI	1.08 (1.04-1.14)

Number of subjects: 1277; Number of events: 344 (26.9%); Number of observations: 11317. Joint modelling survival analysis allows estimation of the time-varying, patient-specific random effect of the endogenous covariate (urea) on an outcome. Joint models were adjusted for baseline variables (age, renal replacement therapy, sequential organ failure assessment, and protein dose randomisation). Effect estimate is for a two-fold increase in time-varying urea ratio, and time-varying presence of acute kidney injury with 95% credible intervals. The hazard ratios were the adjusted hazard ratios associated with a 1-SD increment in the given variable. Values higher than 1 indicate an increased mortality. The values used for standard deviations were as follows: Age, 16.7 years; and SOFA score, 3.7.

Figure E12. Association between time-varying urea and 30-day mortality using a multivariate joint model including time-varying acute kidney injury.

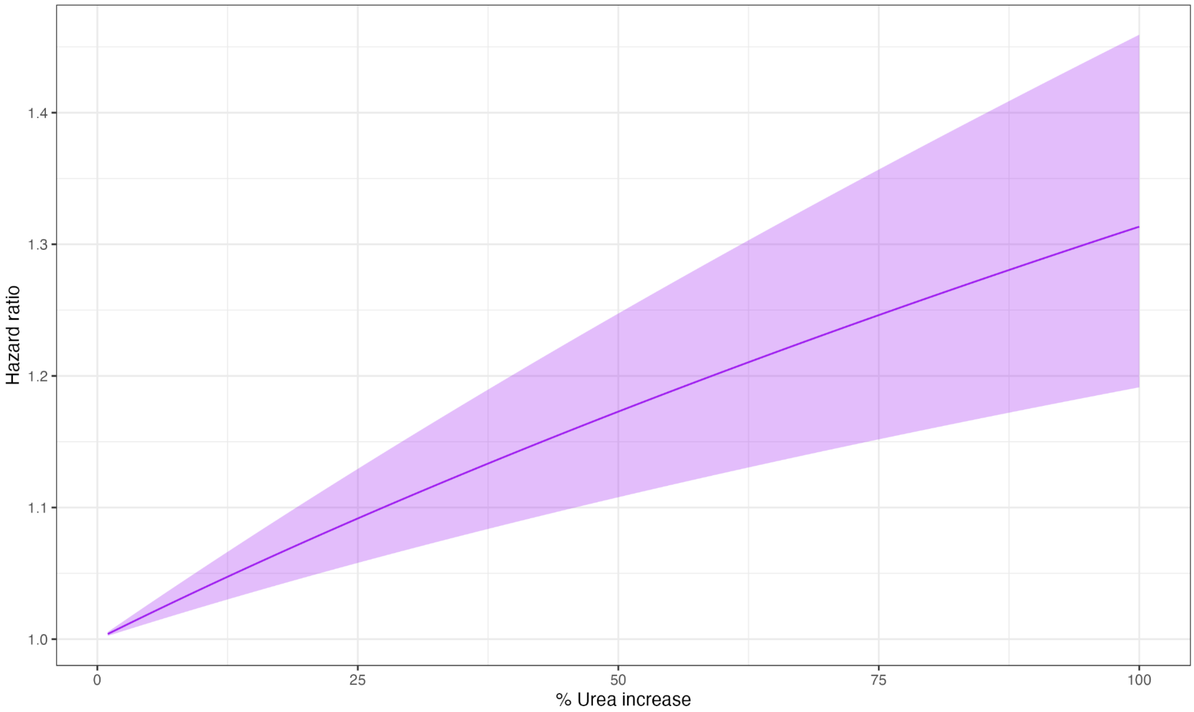

Figure E13. Longitudinal urea-to-creatinine ratio trajectories. Loess with 95% confidence interval.

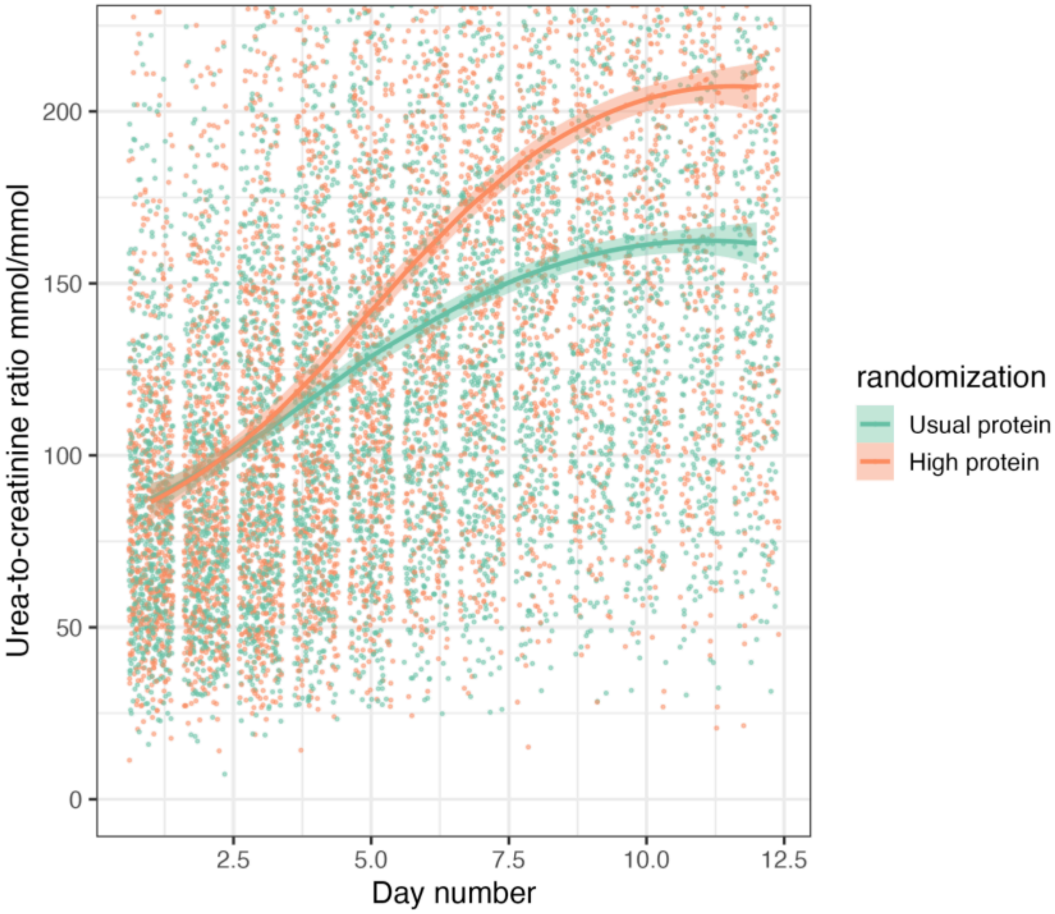

Supplement: Supplementary file 1 — Additional file 1. Online Data Supplement. [file 13054_2024_4799_MOESM1_ESM.pdf]
